# Blood Pressure Response in Miners Exposed to Chronic Intermittent Hypoxia in Chile

**DOI:** 10.3389/fcvm.2021.701961

**Published:** 2021-08-12

**Authors:** Morin Lang, Valeria Paéz, Giacomo Maj, Juan Silva-Urra, Cristián Labarca-Valenzuela, Sergio Caravita, Andrea Faini, Javier Cantuarias, Oscar Perez, Grzegorz Bilo, Gianfranco Parati

**Affiliations:** ^1^Department of Rehabilitation Sciences and Human Movement, Faculty of Health Sciences, University of Antofagasta, Antofagasta, Chile; ^2^Department of Medicine and Surgery, University of Milano-Bicocca, Milan, Italy; ^3^Biomedical Department, Faculty of Health Sciences, University of Antofagasta, Antofagasta, Chile; ^4^Department of Cardiovascular, Neural and Metabolic Sciences, Istituto Auxologico Italiano, Istituto di Ricerca e Cura a Carattere Scientifico (IRCCS), Milan, Italy; ^5^Department of Management, Information and Production Engineering, University of Bergamo, Dalmine, Italy; ^6^Compañia Minera Doña Inés de Collahuasi, Iquique, Chile

**Keywords:** blood pressure, hypertension, ambulatory blood pressure monitoring, chronic intermittent hypoxia, altitude

## Abstract

**Introduction:** Limited information is available on blood pressure (BP) behavior in workers exposed to chronic intermittent hypoxia (CIH), and even less is known regarding effects of CIH on 24-h ambulatory BP in those affected by arterial hypertension at sea level (SL). The aims of this study were to assess clinic and 24-h ambulatory BP at SL and at high altitude (HA; 3,870 m above SL) in workers exposed to CIH, and to compare BP response to HA exposure between normotensive and hypertensive workers.

**Methods:** Nineteen normotensive and 18 pharmacologically treated hypertensive miners acclimatized to CIH were included, whose work was organized according to a “7 days-on−7 days-off” shift pattern between SL and HA. All measurements were performed on the second and seventh day of their HA shift and after the second day of SL sojourn.

**Results:** Compared to SL, 24-h systolic BP (SBP) and diastolic BP (DBP) increased at HA [+14.7 ± 12.6 mmHg (*p* < 0.001) and +8.7 ± 7.2 mmHg (*p* < 0.001), respectively], and SBP nocturnal fall decreased consistently (−4.1 ± 9.8%; *p* < 0.05) in all participants, with hypertensives showing higher nocturnal DBP than normotensives (*p* < 0.05) despite the current therapy. Also, heart rate (HR) nocturnal fall tended to be reduced at HA. In addition, the 24-h SBP/DBP hypertension threshold of ≥130/80 mmHg was exceeded by 39% of workers at SL and by 89% at HA. Clinic HR, SBP, and DBP were significantly higher on the second day of work at HA compared with SL, the increase being more pronounced for SBP in hypertensives (*p* < 0.05) and accompanied by, on average, mild altitude sickness in both groups. These symptoms and the values of all cardiovascular variables decreased on the seventh day at HA (*p* < 0.05) regardless of CIH exposure duration.

**Conclusion:** Long history of work at HA according to scheduled CIH did not prevent the occurrence of acute cardiovascular changes at HA during the first days of exposure. The BP response to HA tended to be more pronounced in hypertensive than in normotensive workers despite being already treated; the BP changes were more evident for 24-h ambulatory BP. Twenty-four-hour ABP monitoring is a useful tool for an appropriate evaluation of BP in CIH workers.

## Introduction

In the last decades, Chile has seen an explosive increase in working activities at high and very high altitude (HA) that is between 2,500 and 5,800 m above sea level (m a.s.l.), especially in the mining facilities located at HA in roughly 80% of cases (1). Miners, in these circumstances, are repeatedly exposed to environmental changes due to the rotating shift system modality, where all individuals alternate periods of work at HA and of rest at low altitude or sea level (SL) for a time proportional to the time worked. Such work organization leads to the so-called chronic intermittent hypoxia (CIH) exposure and is thought to be preferable to continuous HA permanence because of a lesser impact on social and health-related aspects of workers' lives. The most used shift modalities in CIH exposure are 4 days on (at HA) vs. 3 days off (SL) and 7 on vs. 7 off days; the latter being the most widely used and associated with a reasonable quality of social and family life of workers (2).

Acute exposure to hypobaric hypoxia induces several adjustments in the cardiovascular system (3–6), principally related to an increased sympathetic nervous activity, owing to chemoreceptor stimulation by hypoxia (7, 8). This leads to increases in heart rate (HR) and blood pressure (BP) (6, 8, 9), including a significant increase in ambulatory BP over 24 h and in particular at night (10). The long-term adjustments to CIH tend to be similar to those observed for chronic hypoxia exposure, in terms of ventilatory, cardiovascular, erythropoietic, and physiological responses; however, at similar altitude levels, differences have been observed in the time needed to complete the acclimatization, depending on the parameters of interest. Continuous chronic hypoxia requires a few months for acclimatization, while CIH seems to require several years to stabilize some of the acclimatization parameters, while others fail to stabilize even in the long term (11). A cohort study in Chilean miners exposed to CIH showed higher values of mean systemic BP during the day and nighttime compared to SL, with a tendency for BP to decrease with time. Moreover, persisting HA sickness, decreased physical capacity, pulmonary hypertension, and altered sleep patterns have been reported after 31 months of sleeping at 3,800 m and working at 4,800 m, with 7-on−7-off shifts (12).

Previous studies have suggested that individuals with hypertension (especially if uncontrolled) may display more pronounced BP increase when acutely exposed to HA, with a hypoxia-driven enhanced BP response to maximal exercise (13–16). It is not known, however, whether similar responses occur during CIH. In particular, the latter finding may carry some safety concerns, considering that miners perform physical work with a high cardiovascular load, and that in 2017, arterial hypertension was reported to affect 13.8% of the mining population following 7-on−7-off shifts in Chile (17). To date, limited data obtained with ambulatory blood pressure (ABP) monitoring (ABPM) in workers exposed to CIH are available (12), even if ABPM is recognized by international guidelines as an important instrument in the diagnosis and management of hypertension (18, 19), with some evidence indicating its superiority over conventional BP measurement also in the HA setting (6).

Even less is known on BP behavior in hypertensive (HT) patients, as well as on the effectiveness of anti-HT drugs in such conditions. The main aim of this study was thus to assess clinic BP and ABP in normotensive (NT) and HT workers exposed to CIH both during HA permanence and at SL. A secondary objective was to explore the features of BP response in HT workers and to indirectly assess the efficacy of their ongoing anti-HT treatment in this setting.

## Methods

### Design and Participants

In this pilot prospective observational study, we considered the whole employee database of a mining company in northern Chile, located at 3,870 m above sea level (m a.s.l.). In May 2019, 49 male workers who responded positively to the call were enrolled. Subjects were included if they fulfilled the following criteria: age 19–60 years; working in 7-on−7-off shifts since more than 2 years; permanent residence at low (<500 m) altitude; either workers with no hypertension history (NT) or HT workers on anti-HT treatment; and written informed consent to participate in the study. Hypertension diagnosis was based on the screening performed at intervals ranging between 1 and 3 years (depending on age). The exclusion criteria were as follows: HT subjects with no pharmacological treatment; history of serious mountain sickness; cardiovascular diseases other than hypertension (e.g., atrial fibrillation); suspected or confirmed secondary hypertension; diabetes mellitus; other conditions deemed relevant by the investigator; body mass index (BMI) ≥ 35 kg/m^2^; and elevated probability of non-compliance with the study procedures. All participants gave their written informed consent prior to the study. The protocol was approved by the Scientific Research Ethics Committee of the University of Antofagasta (approval number: 181/2019), and the study was conducted in agreement with the Declaration of Helsinki principles.

### Study Organization

In the first phase of the study, the available information was collected on the occupational health of workers who agreed to participate in the study.

**Figure 1:**
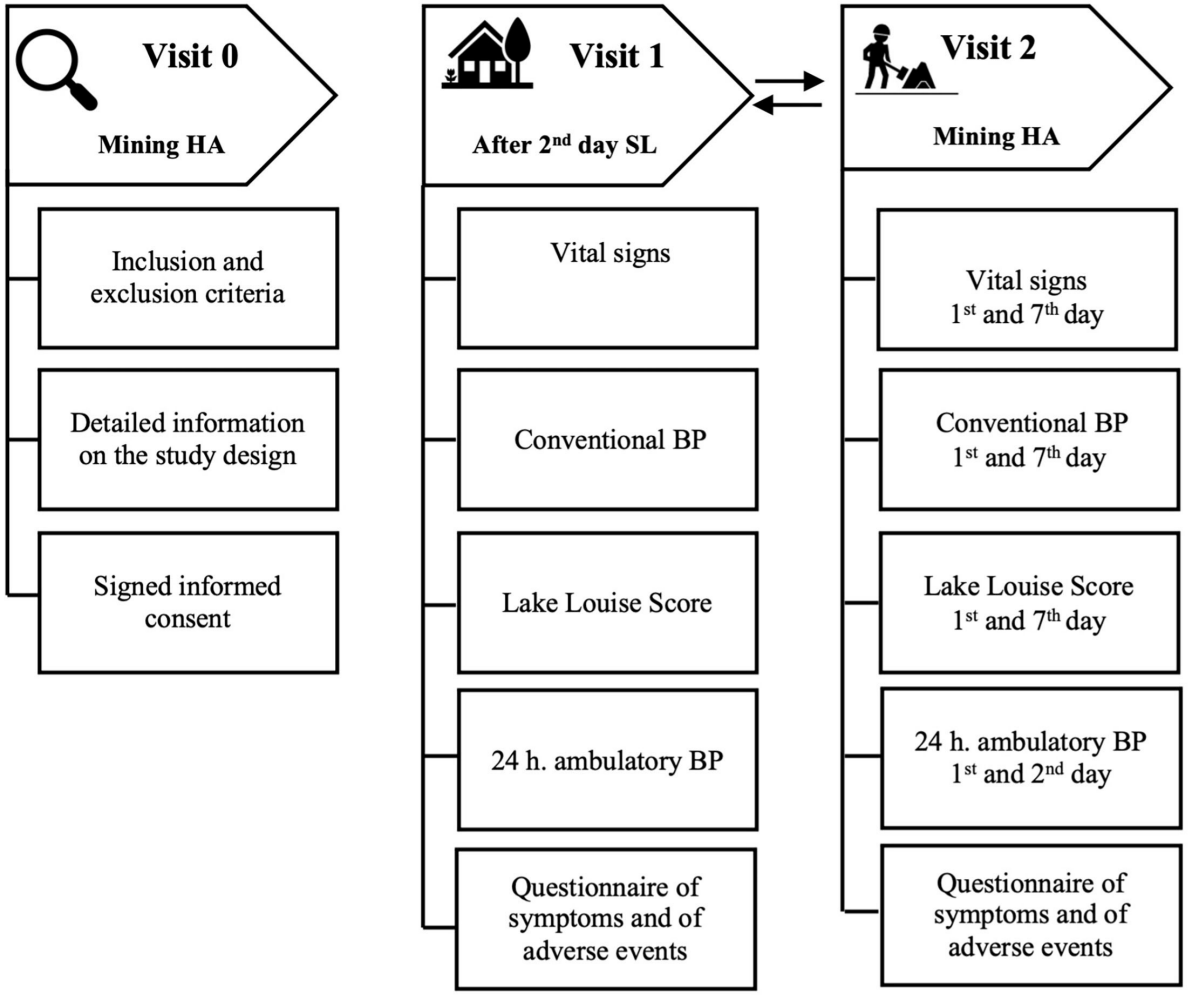
Study design and visits.

Relevant clinical variables and information on symptoms and clinical history, cardiovascular risk factors, history of hypertension, and ongoing anti-HT drug treatment in workers with high BP, and initial verification of inclusion and exclusion criteria were obtained during study visit 0 at mine base camp. The individuals who met the selection criteria at visit 0 continued with the study visits 1 and 2 performed in a random sequence in the following conditions: at SL on any day after at least 2 days of sojourn at SL during their 7 days off from a work shift (visit 1), and at HA during their HA work shift, with data collection on the first, second, and seventh days of exposure (visit 2). During visit 1, the information on symptoms was collected, as well as 24-h ABPM, clinic BP, blood oxygen saturation (SpO_2_), and Lake Louise Score (LLS) of acute mountain sickness (AMS). During visit 2, a questionnaire of symptoms was completed, and clinic BP, LLS, and vital signs were obtained in the early morning, on the first day of a shift after a night spent in the mining dormitory at 3,870 m a.s.l. (day 1). ABP monitor was applied before the participants' ascent to the mining operations site located between 4,203 and 4,797 m a.s.l. on day 1 and removed after 24 h on the second day in the early morning. Finally, on the last shift day (day 7), early morning clinic BP, LLS, and vital signs collection were recorded again. ABPM repetition was foreseen also on day 7 in the study protocol, but it was not performed because it was deemed to interfere with working activities, carrying the risk of work interruptions, and with workers' safety. Thus, the occupational health management of the mining company did not authorize ABPM performance on day 7. The design of the study and the distribution of study visits are shown in Figure 1.

### Measurements

The study measurements (with exclusion of ABPM) were performed at HA in the clinic of the mining camps and at SL in the private homes of each worker, and in the university premises in towns where the participants resided including Arica, Iquique, Antofagasta, Copiapo, La Serena, Viña del Mar, and Santiago.

The following data were collected during the study: clinical history, anthropometric parameters, vital signs (including clinic BP, HR, and SpO_2_), and 24-h ABP.

Clinical history and information on ongoing pharmacological treatment were collected in all participants; moreover, on several occasions, the individuals were asked to report the occurrence of specific signs or symptoms. Significant clinical events detected through this modality could be noted, together with the circumstances in which they occurred. The actual altitude of work was recorded for each participant.

Clinic BP values were measured with validated oscillometric instruments (AND UA-767 Plus, AND, Tokyo, Japan); SpO_2_ and HR were measured on the subject's index finger with a pulse oxymeter (Vantage 9590, NONIN, Plymouth, MN, USA). AMS was diagnosed based on the LLS system (20) and defined as the presence of headache with total LLS score of three or more points from the four rated symptoms (headache, gastrointestinal symptoms, fatigue and/or weakness, and dizziness). AMS was then classified as mild (3–5 points), moderate (6–9 points), or severe (9–12 points) (20). Body height and weight were measured with a validated scale and an altimeter, and waist circumference with a meter tape.

Twenty-four-hour ABPM was performed with a validated oscillometric device (TM-2430, AND, Tokyo, Japan), applied to the non-dominant arm in the morning and removed after 24 h, with subjects instructed to stay immobile during measurements and to attend their usual activities during the recording period while avoiding strenuous exercise. Each monitoring began in the morning, between 7:00 and 11:00, and continued while the worker carried out the normal daily tasks. Measurements were programmed every 15 min during daytime (7–22 h) and every 20 min at night (22–7 h). Mean values were computed for systolic BP (SBP), diastolic BP (DBP), and HR over 24 h, daytime, and nighttime. Nocturnal BP fall was calculated as percent reduction in daytime BP at night. Only recordings with at least 70% of expected readings rated as valid based on predefined criteria were considered.

To investigate the prevalence of high BP values and hypertension, the following definitions were used: (a) clinic hypertension: clinic SBP ≥ 140 mmHg and/or DBP ≥ 90 mmHg; (b) 24-h hypertension: 24-h ambulatory SBP ≥ 130 mmHg and/or DBP ≥ 80 mmHg; (c) daytime hypertension: daytime ambulatory SBP ≥ 135 mmHg and/or DBP ≥ 85 mmHg; (d) nighttime hypertension: nighttime ambulatory SBP ≥ 120 mmHg and/or DBP ≥ 70 mmHg (19).

### Statistical Analysis

Based on the values of the primary outcome variable (24-h ambulatory SBP) obtained in previous studies, at least 36 patients (18 per group) were required to identify a difference of 6 mmHg in the primary efficacy variable between altitude exposure conditions.

Descriptive statistics are presented as means and standard deviations and absolute and relative frequencies overall and separately for non-HT and HT subjects. Shapiro–Wilk test was used for assessing normality in group data. Log transformation was applied to data without normal distribution. Levene's test was used for assessing variance homogeneity. To determine the effects of altitude (3: sea level, SL; high altitude day 1, HA-D1; and high-altitude day 7, HA-D7) and group (2: NT and HT) effects on the primary outcome (24-h ambulatory SBP) and on other variables of interest (other ambulatory BP variables, clinic HR, SBP, DBP, and SpO2), a mixed model multivariate analysis of variance (MANOVA) was applied. The follow-up was carried out by means of univariate analyses contrasts. Finally, in the case of significant effects, pairwise comparisons with Tukey correction were performed. The data were analyzed using the SPSS 25.0 statistical package (SPSS Inc, Chicago, IL, USA). For all statistical tests, an alpha level of 0.05 was used.

## Results

### General Characteristics

Forty-nine subjects who responded positively to the call were voluntarily enrolled. Of these, 12 did not complete the study because of non-compliance with study procedures (data collection at SL could not be completed). Thus, 37 participants were included in the final analysis: 19 in the NT and 18 in the HT group. There were no significant differences in the general characteristics between groups at baseline, except for a higher age in HT group (*p* = 0.02; Table 1). Ongoing anti-HT treatment in the HT group included angiotensin II receptor blockers (nine participants), β-blockers (three participants), ACE inhibitors (two participants), calcium antagonist (one participant), combinations of angiotensin II receptor blockers + calcium antagonist (two participants), and calcium antagonist + β-blocker (one participants). No treatment changes occurred between the SL and HA conditions.

**Table 1:** Demographic, anthropometric, and clinical characteristics of all subjects and by groups (NT and HT).

	**All (*n* = 37)**	**NT (*n* = 19)**	**HT (*n* = 18)**
Age (years)	48.5 ± 6.8	45.8 ± 6.2	51.5± 6.4^*^
Exposure time (years)	10.4 ± 4.3	10.2 ± 5.0	10.6 ± 3.6
BMI (kg/m^2^)	29.3 ± 2.8	30.1 ± 2.3	28.3 ± 3.1
Normal weight (*N* [%])	3 [8.1]	0	3 [16.7]
Overweight (*N* [%])	20 [54.1]	12 [63.2]	8 [44.4]
Obesity (*N* [%])	14 [37.8]	7 [36.8]	7 [38.9]
WC (cm)	101.9 ± 6.8	103.5 ± 6.9	100.1 ± 6.4
WC > 102 cm (*N* [%])	19 [51.4]	11 [57.9]	8 [44.4]
Smokers (*N* [%])	12 [33.3]	7 [36.8]	5 [27.8]
Work altitude (m a.s.l.)	4,459 ± 329	4,436 ± 325	4,482 ± 333
Known obstructive sleep apnea (*N* [%])	5 [13.5]	3 [15.8]	2 [11.1]

*WC, waist circumference*.

* *p < 0.05 for comparison between NT and HT groups*.

### Cardiovascular Variables and AMS

Significant increases in resting clinic SBP and HR from SL were observed on day 1 of HA exposure for all participants. On day 7, SBP tended to return to SL values, while HR decreased but remained higher than at SL (*p* < 0.001) (Table 2). Compared to SL values (97.0 ± 0.5%), SpO_2_ significantly decreased on the first day at HA (88.0 ± 3.2%; *p* < 0.001) and remained significantly lower on the seventh day (88.4 ± 5.5%; *p* < 0.001) in all participants. Mild AMS occurred in 16.2% of participants on the first day at HA (*p* < 0.001) with no cases of severe AMS, decreasing to 0% on the seventh day (Table 2). A significant difference between HT and NT participants was found in SBP at SL, on the first and seventh days at HA and in DBP at SL. However, no significant interaction was found for group × altitude effect [*F*_(8;25)_ = 0.323; *p* = 0.949], while a significant effect of altitude [*F*_(8;25)_ =50.01; *p* < 0.001] was observed.

**Table 2:** Clinic systolic blood pressure (cSBP), clinic diastolic blood pressure (cDBP), HR, SpO_2_ at SL, and AMS prevalence on the first and seventh days at HA in all participants and grouped by NT and HT condition.

	**Group**	**SL**	**HA-D1**	**HA-D7**
cSBP (mmHg)	NT	115.74 ± 10.3	123.47 ± 13.0	116.47 ± 7.0
	HT	125.20 ± 11.9^Δ^	133.77 ± 15.2^Δ^	124.16 ± 17.1^Δ^
	ALL	120.20 ± 11.9	128.48 ± 14.9^§^	120.21 ± 13.4
cDBP (mmHg)	NT	73.37 ± 8.1	78.74 ± 9.9	78.32 ± 9.2
	HT	81.26 ± 7.5^Δ^	83.64 ± 9.5	84.05 ± 9.8
	ALL	77.09 ± 8.7	81.05 ± 9.9	81.02 ± 9.8
HR (bpm)	NT	64.71 ± 9.1	81.36 ± 18.2	73.95 ± 11.2
	HT	62.76 ± 10.7	81.60 ± 15.1	75.66 ± 12.2
	ALL	63.79 ± 9.8	81.38 ± 15.8^*^	74.78 ± 11.5^*^
SpO_2_ (%)	NT	97.76 ± 0.6	88.00 ± 3.3	89.26 ± 5.3
	HT	97.91 ± 0.4	88.05 ± 3.2	87.5 ± 5.8
	ALL	97.83 ± 0.5	88.02 ± 3.2^*^	88.40 ± 5.5^*^
AMS count; (%)	NT	0; (0)	5; (26.3)^§^	0; (0)
	HT	0; (0)	1; (5.5)	0; (0)
	ALL	0; (0)	6; (16.2)^§^	0; (0)

*Data expressed as mean ± standard deviation*.

* *Statistically significant differences between SL and all HA conditions*.

§ *Statistically significant differences between SL and HA-D1*.

*^∧^Statistically significant differences between HA-D1 and HA-D7*.

Δ *Statistically significant differences between HT and NT groups*.

### Twenty Four Hour ABPM Parameters

As shown in Table 3, HR, SBP, and DBP values over 24 h, daytime, and nighttime increased significantly at HA compared with SL values in the entire sample (*p* < 0.001). Twenty-four-hour SBP and 24-h DBP increased by 14.7 ± 12.6 mmHg (*p* < 0.001) and 8.7 ± 7.2 mmHg (*p* < 0.001), respectively, and HR increased by 14.7 ± 9.0 beats/min (Table 3). Table 4 shows the SBP, DBP, and HR for 24 h, daytime, nighttime as well as nocturnal fall values by visit (SL-HA) and by subgroup (NT and HT). SBP, DBP, and HR showed a significant increase over 24 h, daytime, and nighttime. The magnitude of 24-h ambulatory BP increases at HA was numerically less in the NT group than in the HT group (11.3 ± 12.6 mmHg NT vs. 18.46 ± 11.7 mmHg on HT for SBP; and 7.05 ± 7.7 mmHg NT vs. 10.5 ± 6.3 mmHg HT for DBP, respectively), but these differences did not reach the level of statistical significance (Table 4). No significant differences were observed in 24-h ABPM parameters at SL between groups (Table 4). A borderline significant difference between groups was observed in nocturnal values of SBP (*p* = 0.06) and DBP (*p* = 0.04) (Table 4). Furthermore, the nocturnal fall of BP in individuals belonging to the HT subgroup tended to decrease in a more pronounced manner than in the NT group with nocturnal fall of DBP being, on average, within the “non-dipper” range (<10%), but the observed differences between groups did not reach statistical significance (Table 4).

**Table 3:** HR and BP variables from 24-h ABPM at SL and HA.

**Variable**	**SL**	**HA**	***p***
**24-h**			
SBP	124.8 ±10.9	139.5 ± 12.6	<0.001
DBP	74.6 ± 6.9	83.4 ± 8.0	<0.001
HR	68.7 ± 7.8	83.4 ± 10.3	<0.001
**Day**			
SBP	130.7 ± 11.6	143.5 ± 13.1	<0.001
DBP	78.3 ± 6.8	85.8 ± 8.6	<0.001
HR	72.3 ± 8.1	85.8 ± 10.1	<0.001
**Night**			
SBP	111.3 ± 12.5	128.0 ± 16.3	<0.001
DBP	66.3 ± 8.1	75.9 ± 8.9	<0.001
HR	60.8 ± 7.8	75.9 ± 13.2	<0.001
**Nocturnal fall**			
SBP	19.5 ± 11.1	15.5 ± 14.3	0.114
DBP	12.0 ± 5.9	9.9 ± 7.7	0.164
HR	11.5 ± 4.9	9.9 ± 8.8	0.331

*All BP values in mmHg and HR values in bpm. Nocturnal fall as % of daytime values*.

*Data expressed as mean ± standard deviation*.

**Table 4:** Mean 24-h, daytime, nighttime, dipping and work SBP, DBP, and HR at SL and HA by groups (NT and HT).

**Variable**	**Visit**	**NT**	**HT**	**Visit NT**	**Visit HT**	**NT-HT comparison**
				**(** ***p*** **)**	**(** ***p*** **)**	**(** ***p*** **)**
24 h SBP	SL	126.2 ±10.5	123.3 ± 11.5	0.001	<0.001	0.437
	HA	137.6 ± 11.2	141.8 ± 13.7			0.306
DBP	SL	74.5 ± 6.4	74.8 ± 7.6	0.001	<0.001	0.916
	HA	81.6 ± 7.5	85.3 ± 8.1			0.159
HR	SL	69.5 ± 7.4	67.8 ± 8.3	<0.001	<0.001	0.522
	HA	84.6 ± 9.8	83.0 ±11.2			0.640
Day SBP	SL	132.7 ± 11.4	128.6 ± 11.9	0.014	<0.001	0.299
	HA	141.8 ± 12.3	145.3 ± 13.7			0.412
DBP	SL	78.3 ± 6.7	78.31 ± 7.1	0.006	<0.001	0.971
	HA	84.0 ± 7.9	87.8 ± 8.9			0.198
HR	SL	73.1 ± 8.1	71.3 ± 8.3	<0.001	<0.001	0.513
	HA	87.3 ± 9.2	84.9 ± 11.2			0.472
Night SBP	SL	110.6 ± 11.8	112.0 ± 13.5	0.001	<0.001	0.747
	HA	123.5 ±10.5	133.2 ± 19.5			0.064
DBP	SL	65.4 ± 6.7	67.3 ± 9.4	0.004	<0.001	0.491
	HA	73.3 ± 7.9	79.2 ± 9.1			0.044^*^
HR	SL	61.1 ± 6.4	60.5 ± 9.2	<0.001	<0.001	0.829
	HA	75.2 ± 13.8	76.1 ± 12.4			0.685
NF (%) SBP	SL	22.1 ± 11.0	16.6 ± 10.7	0.263	0.285	0.141
	HA	18.3 ± 11.0	12.1 ± 16.7			0.187
DBP	SL	12.9 ± 6.2	11.0 ± 10.6	0.310	0.365	0.323
	HA	10.7 ± 6.5	8.4 ± 9.1			0.392
HR	SL	12.1 ± 4.9	10.8 ± 5.0	0.964	0.101	0.455
	HA	12.2 ± 9.6	7.9 ± 7.3			0.142

*NF, nocturnal fall % of daytime values*.

*Data expressed as mean ± standard deviation*.

* *Statistically significant differences between NT and HT groups*.

**Figure 2:**
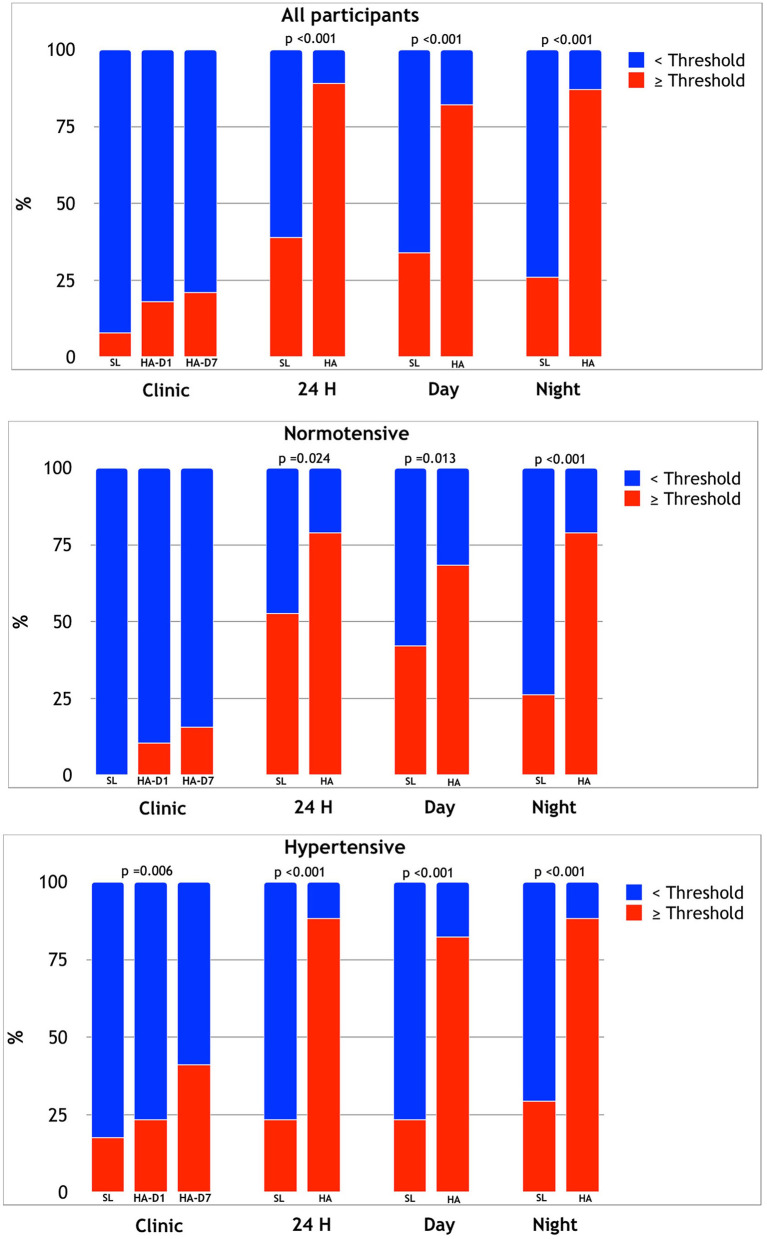
Relative prevalence of participants exceeding clinic, mean 24-h, daytime, and night HT threshold at SL and HA in the overall sample (upper panel), in NT (middle panel), and HT (lower panel) subjects.

For descriptive purposes, categorical analysis was performed classifying participants according to BP thresholds for hypertension diagnosis and assessing hypertension control in treated participants (Figure 2). The percentage of participants with elevated clinic BP was generally low at SL (8% of the overall sample) and increased to about 20% at HA (*p* = 0.05) with most of the subjects (both NT and HT) remaining below hypertension/uncontrolled hypertension threshold. When considering 24-h ABPM, a considerable proportion of subjects had mean values above the diagnostic thresholds, and during HA exposure, the vast majority of both NT and HT subjects had mean 24-h, daytime, and nighttime values above the thresholds for hypertension/uncontrolled hypertension (up to 88% for 24-h average SBP or DBP in HT patients; Figure 2).

## Discussion

Our study provides novel information on the conventional and ambulatory BP responses to CIH in a group of miners acclimatized to this condition, including HT individuals. To this aim, workers were evaluated during their working shift at HA by clinic BP and by ABPM and subsequently during the days off in their homes at SL. We show that miners, despite being acclimatized to CIH (according to Chilean Ministry of Health a worker is considered acclimatized to chronic intermittent hypobaria if he/she has been working for more than 6 months in rotating shifts at HA and resting at low altitude), exhibit a significant acute response in cardiovascular variables, including a significant increase in clinic and 24-h ambulatory BP and HR in the first days of shift work at HA. Compared to NT individuals, this increase tended to be more accentuated in HTs hypertensive participants (especially during the night), despite treatment. Moreover, at HA, most workers exceeded the diagnostic thresholds of hypertension in 24-h ABPM. The information obtained with 24-h ABPM represents the main element of novelty in our study and is of particular importance, considering the established advantages of ABPM over conventional clinic BP measurements (6), also when exploring the environmental effects on BP (9, 10, 16). The individuals included in this study presented the general characteristics typical of those who work in Chile and are exposed to CIH for a long period. The miners in this study had on average high BMI values, displaying a high prevalence of overweight and obesity, as described previously (21, 22). Either in terms of overweight/obesity and in terms of smoking prevalence, our sample is comparable with the male population of Chile according to the 2016–2017 Health National Survey (23). The workers in our study had a history of CIH exposure lasting in some cases over 10 years and spent the 7 days of the work shift at an altitude between 3,800 m a.s.l. (the location of the dormitories), and higher altitudes up to roughly 4,800 m, reached daily by the miners for working activities. We included subjects working at HA since at least 2 years because of previous evidence indicating that the acclimatization to CIH occurs after 18 months (12). Most of the workers reached an altitude >4,203 m a.s.l. daily; of these, just less than a half reached the extreme altitude of more than 4,797 m a.s.l. daily. This type of intermittence and altitude reached is in line with previous definition of “chronic intermittent hypoxia” (12, 24).

### Twenty-Four-Hour ABP

The use of ABPM to study BP responses to hypoxia represents a novel approach in the specific field of investigation on the effects of CIH exposure at HA. This technique is known to provide more solid information on real BP levels in daily life conditions than clinic BP, and its usefulness was confirmed also in HA research (6). In the only previous study using ABPM in CIH workers, this technique was only used at HA (12). In our study, we show that in NT individuals as well as in treated HT patients, exposure to CIH at HA induces a significant BP increase over 24 h, daytime, and nighttime, accompanied by a tendency to a reduced nocturnal dipping of BP, in line with our previous findings during acute exposure (16). The reduction in nocturnal BP and HR dipping could be due to a greater reduction in SpO_2_ during sleep at HA (10, 16); however, we could not verify this hypothesis in the present study.

NT and treated HT workers had similar ambulatory BP mean levels at SL, indicating that HT patients were generally well-controlled by treatment in this condition; however, at HA, the nocturnal BP levels were significantly higher and the degree of dipping in HT patients tended to be more frequently abnormal, as compared to NT individuals. In particular, HT participants displayed a significantly larger increase in nocturnal BP, accompanied by abnormally reduced nocturnal BP fall (on average <10%), as compared to NT individuals. Such nocturnal BP patterns are associated with increased cardiovascular risk in lowlanders (25, 26). Regarding treatment coverage of HT patients at SL, it must be noted that while in almost all participants clinic BP values were well-controlled, in some of them ambulatory BP was elevated. This so-called masked uncontrolled hypertension phenotype was even more frequent during HA exposure. This confirms the importance of ABPM in determining the real effectiveness of an anti-HT antihypertensive therapy over 24 h, especially in relatively young individuals in whom work-related BP elevation may have a considerable impact on overall BP burden (16, 27–29).

### Clinic BP and Other Variables

Our results are in accordance with previous findings that described, compared with SL, a BP and HR increase, a SpO_2_ decrease, and a certain incidence of altitude sickness on the first day after arrival at altitude (12, 30). The incidence of AMS on day 1 in our study agrees with the recent data reported by Chilean Superintendence of Social Security (17). It appears that prolonged exposure to CIH does not fully prevent the appearance of an acclimatization response associated with acute exposure to altitude. In our study at the end of the HA shift, clinic BP reached pre-exposure values and HR decreased despite persistently reduced SpO_2_. These results differ from what was found by Brito et al. (30), who reported a rapid normalization of these parameters from the second day, but without reaching SL values. However, this study was carried out at a lower altitude and made use of a different shift system. Our study was not designed to investigate the underlying mechanisms of the changes we observed. However, it may be hypothesized that as time passes at altitude, a new balance between peripheral hypoxic vasodilation and sympathetic-driven vasoconstriction is established, possibly involving a resetting of peripheral chemoreflex modulation. This generates a drop in arterial pressure, with a minor effect on the HR. Such a dissociation between the BP and HR responses (31) is associated with persistently low SpO_2_ values found in the workers at the end of the shift. In agreement with the present results, previous studies have demonstrated increased HR persisting even after acclimatization at HA (32, 33).

The increase in clinic BP on the first day of exposure to altitude in CIH appears similar to what occurs during an acute HA exposure in unacclimatized lowlanders and seems to depend on the predominance of sympathetic-mediated pressor mechanisms, mainly triggered by hypoxia-induced chemoreflex stimulation (6, 34), prevailing over direct vasodilatory effect of hypoxia. In fact, earlier observations showed a pressor response both in NT (10) and HT lowlanders (16) exposed acutely to HA, and in NT (30) and HT subjects (35) after long-term exposure to CIH. Clearly, other factors, not quantified in our study, could have contributed to the observed changes. In particular, work-related physical, and mental stress could be relevant. However, we believe that its relevance was limited, considering that (1) the jobs of included participants (operators, watchmen, electricians, draftsmen, mechanics, warehouse workers, and supervisors) do not have a high physical workload, and (2) the impact of both physical and mental stress is unlikely to explain the observed increase in nocturnal BP.

Overall, the present findings seem to be consistent with other studies on miners and soldiers exposed to long-term intermittent hypoxia, which found an increase in the proportion of individuals reaching elevated BP values, exceeding in many cases hypertension threshold (11, 22, 30), a phenomenon that was particularly pronounced in those with a previous diagnosis of hypertension (35). Vinnikov et al. showed that after 1 year of intermittent exposure to hypobaria, hypoxia did not lead to BP increase; however, in this study, BP was only assessed at SL (36).

Our findings have important implications for developing preventive programs for occupational health management and health surveillance by indicating the importance of 24-h ABPM during high-altitude work. This may be especially true in HT workers with elevated cardiovascular risk, given that hypertension is the main risk factor for cerebrovascular disease (CVD) and coronary heart disease (37). In addition, miners are exposed to a greater work-related cardiovascular load, which, in a condition characterized by hypoxia, may trigger myocardial ischemia even in the presence of subcritical coronary lesions (13, 38).

Of note, in our study, the participants were assessed on a working day at HA and on a rest day at SL. In our view, this represents a strength of the study rather than its limitation. In fact, with this approach, we were able to explore BP responses to the real-life condition these workers are exposed to, rather than to a specific physiological stimulus such as hypoxia. Therefore, our results may directly apply to HA workers who follow this kind of work model.

## Limitations of the Study

We have to acknowledge a few limitations of our study. First of all, considering the difficult organization of the study due to the challenging environment conditions and to the compromise necessary to address the requirements imposed by work organization in this HA mine as well as by miners' preferences, our sample size was limited; while the number of participants was sufficient for the principal analyses, many pairwise comparisons did not reach statistical significance in *post hoc* comparisons. Some degree of selection bias due to voluntary participation could also have been possible. However, we believe that our sample was fairly comparable with the overall population of Collahuasi miners given that BMI was similar (29 kg/m^2^) and mean age was only slightly higher (48.5 vs. 42 years), according to the available information. We cannot exclude the presence of some residual confounding by factors such as undiagnosed sleep apnea (although the prevalence of known sleep apnea was low). Moreover, since treatment was not standardized, we could not specifically explore the efficacy of any given antihypertensive agent and the possible differences between different antihypertensive drug classes in terms of their efficacy in controlling BP at HA. We included exclusively male participants. However, given that the vast majority of individuals exposed to CIH are men (91.55%) (39), this appears to be a minor limitation, which does not prevent our results from being relevant to the population of HA miners at large. Miners' activity was different at HA (working days) and at SL (off days), with a possible confounding effect on daytime BP. However, the observed differences in nighttime BP values support the major role of HA hypoxia in this regard, independently from differences in behavioral activities. Furthermore, because of mine company worries about possible interference with miners' work, a second 24-h ABPM over 24 h initially planned on the seventh day of HA exposure could not be performed. Finally, the accuracy of oscillometric devices at HA is largely unknown, even if our unpublished data indicate that it is not meaningfully affected (40).

## Conclusion

Miners exposed to CIH display an acute response in cardiovascular variables during the first days at HA despite a history of exposure to CIH exceeding in some cases 10 years. The BP response to HA hypoxia tended to be more accentuated in treated HT individuals, despite BP values at SL similar to those of NT subjects. Twenty-four-hour ABPM confirmed its usefulness as a tool for a better BP burden assessment than that offered by conventional measurements, allowing to identify a considerable proportion of participants with masked uncontrolled hypertension and to detect nocturnal hypertension, associated with a non-dipper 24-h BP profile. This technique could thus be applied in HA workers exposed to CIH in order to better assess and control their cardiovascular risk, without majorly interfering with their work performance, based on our data. Considering that HA workers spend a considerable amount of time in this condition, an increased HA-related BP burden might represent a relevant risk factor. However, outcome data in this regard are missing, and thus, strong recommendations on BP management in this setting cannot be issued. Nonetheless, considering that guidelines support prompt normalization of elevated BP in HT patients, an accurate BP monitoring and a well-timed antihypertensive treatment adjustment could be reasonable in this setting, especially in high-risk workers and in those showing pronounced BP elevations at HA.

## Data Availability Statement

The original contributions presented in the study are included in the article/supplementary material, further inquiries can be directed to the corresponding authors.

## Ethics Statement

Study protocol was reviewed and approved by Comité de ética de investigación científica Universidad de Antofagasta. The participants provided their written informed consent to participate in this study.

## Author Contributions

ML, GB, and GP contributed to conception and design of the study. ML achieved the acquisition of funds for this project. ML, GB, VP, CL-V, JC, OP, and JS-U were mostly implicated in logistics and development of the experimental work at sea level and high altitude. ML and GB led the writing of the manuscript. ML, GB, and GM performed the data curation and statistical analysis. ML, GB, GP, VP, GM, SC, AF, JC, and OP contributed to the interpretation of the results and provided critical feedback on drafts, helping the final version of this manuscript. All authors contributed to the article and approved the submitted version.

## Conflict of Interest

JC and OP at the time of the study were employed by the company Compañia Minera Doña Inés de Collahuasi. The remaining authors declare that the research was conducted in the absence of any commercial or financial relationships that could be construed as a potential conflict of interest.

## Publisher's Note

All claims expressed in this article are solely those of the authors and do not necessarily represent those of their affiliated organizations, or those of the publisher, the editors and the reviewers. Any product that may be evaluated in this article, or claim that may be made by its manufacturer, is not guaranteed or endorsed by the publisher.
